# Dissipation Residue Behaviors and Dietary Risk Assessment of Boscalid and Pyraclostrobin in Watermelon by HPLC-MS/MS

**DOI:** 10.3390/molecules27144410

**Published:** 2022-07-09

**Authors:** Le Lv, Yue Su, Bizhang Dong, Wang Lu, Jiye Hu, Xiaolu Liu

**Affiliations:** School of Chemistry and Biological Engineering, University of Science and Technology Beijing, Beijing 100083, China; lvle@ustb.edu.cn (L.L.); suyue_2022@163.com (Y.S.); dongbizhang@ustb.edu.cn (B.D.); 18810861030@163.com (W.L.); jyhu@ustb.edu.cn (J.H.)

**Keywords:** boscalid, pyraclostrobin, watermelon, MRL establishment, dietary risk assessment

## Abstract

Fungicides containing active ingredients of boscalid and pyraclostrobin have been widely applied in watermelon disease control. To provide data for avoiding health hazards caused by fungicides, we investigated its terminal residues and evaluated the dietary risk. In this work, watermelon samples were collected from field sites in six provinces and analyzed with high-performance liquid chromatography-tandem mass spectrometry (HPLC-MS/MS). The average recoveries of boscalid and pyraclostrobin in the watermelon matrix were 97–108% and 93–103%, respectively, with the relative standard deviations (RSDs) ≤ 9.1%. The limits of quantifications (LOQs) were 0.01 and 0.005 mg/kg for boscalid and pyraclostrobin. Twenty-one days after applying the test pesticide with 270 g a.i./ha, the terminal residues of boscalid and pyraclostrobin were all below 0.05 mg/kg and below the maximum residue limits (MRLs) recommended by European Food Safety Authority (EFSA). According to the national estimated daily intake (NEDI), the risk quotients (RQs) of boscalid and pyraclostrobin were 48.4% and 62.6%, respectively. That indicated the pesticide evaluated in watermelon exhibited a low dietary risk to consumers. All data provide a reference for the MRL establishment of boscalid in watermelon for China.

## 1. Introduction

Watermelon (Citrullus lanatus), which belongs to Cucurbitaceae, is widely cultivated and consumed. The mineral and nutritional compositions of watermelon include crude fiber, flavonoids, copper, calcium, and zinc [1]. However, fungal infections, especially grey mold, on watermelons can cause serious food and economic losses [2]. As a fungal disease usually caused by Botrytis cinerea, grey mold is globally distributed on watermelon and other fruits during harvesting [3]. Decayed fruits even produce various toxins, such as aflatoxins and zeaxanthin, causing economic losses and health hazards [4].

**Figure 1:**
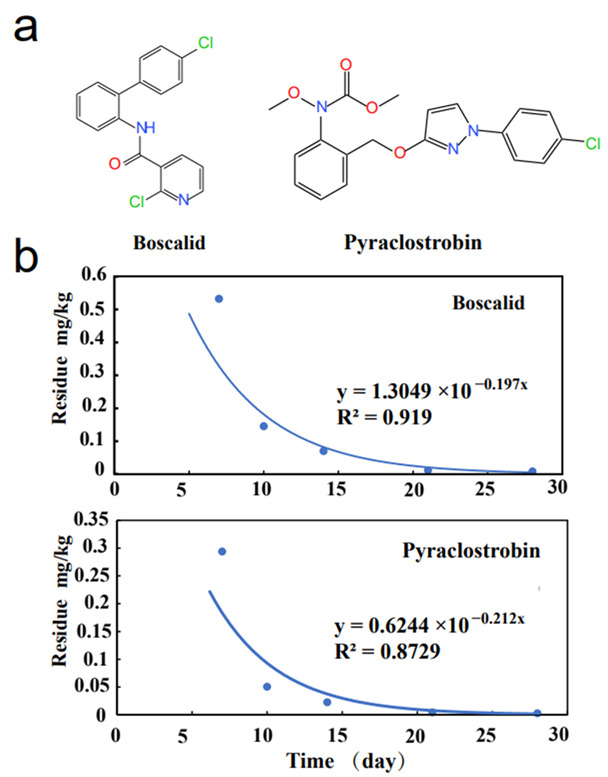
(**a**) Chemical structures and (**b**) half-lives of boscalid and pyraclostrobin in watermelon in Yueyang city of Hunan province.

Fungicides can effectively control fungal diseases and are widely used in practical production. In past years, Pristine^®^ (boscalid + pyraclostrobin) has always been applied as the fungicide for grey mold on watermelons [5]. Boscalid and pyraclostrobin, the active compounds in this fungicide, both exhibit a highly effective antifungal effect. Pyraclostrobin (methyl 2-[1-(4-chlorophenyl) pyrazol-3-yloxymethil]-N-methoxycarbanilate) is a broad-spectrum fungicide of the strobilurin family that inhibits electron transport chain (ETC) CIII activity (Figure 1a) [6]. Boscalid, 2-Chloro-N-(4’-chloro-[1,1’-biphenyl]-2-yl) nicotinamide, is a succinate dehydrogenase inhibitor (SDHI) fungicide that acts on different stages of fungal development (Figure 1a) [7].

However, these fungicides can leave residues after application, where the half-life of degradation (DT50) of boscalid is up to 297–337 days in sandy soil [8]. Correspondingly, the long retention time can give rise to ecological harm. There is evidence of the toxicity induced by boscalid on zebrafish, including changes in cell apoptosis and lipid metabolism, mitochondrial dysfunction, and respiratory impairment [9,10]. Exposure to honeybees via boscalid contaminated water, pollen, and nectar leads to a decline in median time to death (LT_50_) [11]. The ingestion of pyraclostrobin-containing food by mammals, for example, mice, causes adverse health outcomes [12]. Thus, the detection of fungicide residues in fruit is necessary to avoid corresponding health hazards.

Up to now, the residues of boscalid and pyraclostrobin have been detected in grapes [13], citrus fruits [14], and carrots [15]. In previous studies, the LC-MS/MS, RRLC-QqQ-MS/MS, and HPLC techniques were usually adopted in the detection of pesticides in fruits for different matrix characteristics. However, there is still no research that has detected the residues of boscalid and pyraclostrobin at the same time. In addition, the standard maximum residue limits (MRLs) of boscalid in watermelon are not set in China. Thus, this work aimed (1) to establish a sensitive and reliable detection method using QuEChERS and HPLC-MS/MS to quantify boscalid and pyraclostrobin simultaneously, (2) to investigate the terminal residues of these two compounds in watermelon samples from six locations with different climate types in China, and (3) to evaluate the dietary risk of boscalid and pyraclostrobin and to provide data for the establishment of MRL of boscalid in watermelon for China.

## 2. Results and Discussion

### 2.1. Optimization of Instrument Conditions, Extraction, and Purification

**Figure 2:**
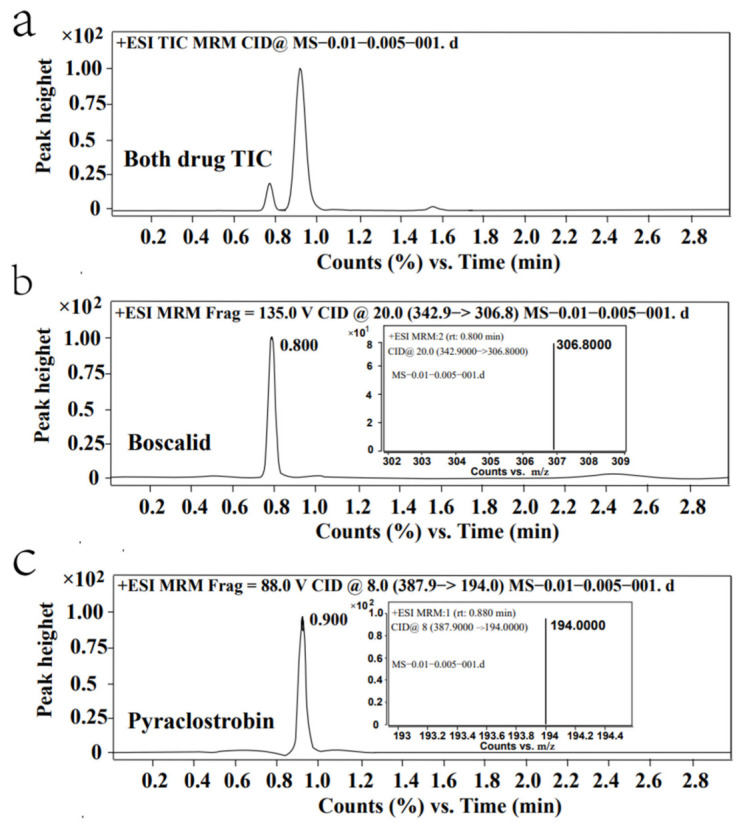
Chromatograms and mass spectra of boscalid and pyraclostrobin; (**a**) show the retention time of boscalid and pyraclostrobin for simultaneous detection; (**b**,**c**) show the chromatographic peaks and quantitative ions of boscalid and pyraclostrobin in MRM mode, respectively.

MS/MS analyses of boscalid and pyraclostrobin were performed in multiple reaction monitoring (MRM) mode and using the acquisition parameters shown in Table S1. The retention times of boscalid and pyraclostrobin were 0.8 min and 0.9 min, respectively, which shortened the detection time and increased the work efficiency (Figure 2). Two product ions were selected as a qualitative ion and a quantitative ion for further optimization. The ions at *m*/*z* 306.8 and 139.9 were the quantitative and qualitative ions of boscalid, respectively, and the ions at *m*/*z* 194 and 163 were the quantitative and qualitative ions of pyraclostrobin (Figure 2 and Figure S2).

Sample preparation is considered an important stage before HPLC-MS/MS. Thus, acetonitrile used as an extraction solvent of boscalid and pyraclostrobin in this study was optimized. In a previous study, four extraction solvents—acetonitrile, methanol, ethyl acetate, and dichloromethane—were compared, and the best recoveries of boscalid, 93.11% and 94.42% in grape and soil, respectively, were extracted by acetonitrile [16]. The average recoveries of boscalid were from 96.4% to 99.0% and from 90.2% to 101.4% for soil and cucumber, respectively, with acetonitrile extraction solvent [17]. 

There are plenty of studies with acceptable recoveries of pyraclostrobin extracted by acetonitrile in various crops or fruits, which were 80.30% to 89.17% in grape, 85% to 102% in wheat, and 75.48% to 103.58% in banana [18,19,20]. In this study, the average recoveries of boscalid and pyraclostrobin in the watermelon matrix were 97–108% and 93–103%, respectively, with RSD ≤ 9.1%. Therefore, acetonitrile or acetonitrile buffer is considered the optimal extraction solvent of both boscalid and pyraclostrobin in this study.

The dispersive soil phase extraction (dSPE) method was adopted in the purification procedure in this study. In general, four common sorbents—PSA, GCB, C18, and MWCNTs—were selected according to different matrices. PSA can sorb various polar matrix components in extraction solutions such as sugars, fatty acids, organic acids, and polar pigments. GCB is able to remove carotenoids and chlorophyll, while C18 is commonly used for the removal of non-polar and medium-polar matrix components. As a result of the costs and the abundant glucose, lycopene, and L-citrulline in watermelon, PSA and GCB were selected to remove the co-extractives in this study. The sorbents, 30 mg of PSA and 5 mg of GCB, gave an acceptable result that shows effective purification and satisfactory recoveries of boscalid and pyraclostrobin in watermelon.

### 2.2. Method Validation

In this study, linearity, sensitivity, precision, and accuracy were assessed to validate the analytical method based on the guideline on pesticide residue trials. The presence of matrix co-extracts after sample preparation frequently affects the signal response in a detector, which is known as “matrix effects” [21]. The matrix effect may interfere with the accuracy of the analytical method in measuring the quantification of the analyte, which causes errors in quantitative or qualitative data, leading to a false-negative or false-positive result [22]. Since complete elimination of the matrix effect is difficult in multi-residue analyses, the matrix-matched standard calibrations were used to calibrate possible interferences in the quantification of the analytes as a compensatory strategy for matrix effects [23]. A blank watermelon (drug-free) sample was added to acetonitrile and cleaned up to prepare a matrix solution. Boscalid and pyraclostrobin were quantitatively added to the matrix solution to prepare 5 different concentrations of the standard matrix solution. A calibration curve was constructed by plotting the corresponding peak areas versus concentrations for the analytes. Satisfactory linearity of the matrix-matched standard calibrations was obtained with the correlation coefficients (r) higher than 0.9900 in the ranges of 0.01–10 mg/kg and 0.005–5 mg/kg for boscalid and pyraclostrobin in the watermelon, respectively (Table S2).

An evaluation of the recoveries and relative standard deviations (RSDs) of boscalid and pyraclostrobin in the watermelon was performed to validate the HPLC-MS/MS method by spiking samples at different levels (Table 1). The recovery (extraction efficiency) was calculated by dividing the peak area of an analyte from a pre-extraction spiked sample by the peak area of an analyte from a post-extraction spiked sample [24]. The method accuracy could be evaluated by recoveries with five replications (*n* = 5) at every concentration. As shown in Table 1, the average recoveries of boscalid and pyraclostrobin in the watermelon matrix were 97–108% and 93–103%, respectively, with the RSDs ≤ 9.1%. According to the provisions of the Guideline on Pesticide Residue Trials (NY/T 788-2018) published by the Ministry of Agriculture, P. R. China, the recovery rate should be 70–120%, RSD ≤ 20% [25]. The limits of quantifications (LOQs) were defined as the lowest spiked concentrations of target analytes in the matrix with a signal-to-noise ratio of 10 [23]. In the conditions mentioned previously, the LOQs were 0.01 and 0.005 mg/kg for boscalid and pyraclostrobin in the watermelon matrix (Figure S1).

In recent years, there have been different methods for detecting boscalid and pyraclostrobin residues on crops. Although some studies choose to develop other methods due to the costs, the main trend of drug residue detection methods is LC-MS/MS, which can detect residues in a variety of crops with high adaptability and high accuracy (Table 2). However, in recent studies, very few methods have been developed to detect the two drugs together [13,26]. Single-drug assays are easier to develop because of their simpler instrument development methods [27,28,29,30,31,32]. Therefore, this study developed an LC-MS/MS technique that can detect boscalid and pyraclostrobin simultaneously, which optimized the drug retention time and greatly shortened the detection time.

### 2.3. Dissipation Behaviors of Boscalid and Pyraclostrobin in Watermelon

The dissipation curves of fungicides in Yueyang city of Hunan province are shown in Figure 1. The curves indicate that the dissipation of boscalid and pyraclostrobin in watermelon followed first-order kinetics. From Table 3, the average residues of the two fungicides are lower than 0.05 mg/kg in the matrix 7 days after application at the other three locations. In this region, the half-lives of boscalid and pyraclostrobin on watermelon were 3.52 d and 3.27 d, respectively. The half-lives of boscalid and pyraclostrobin were reported to vary in different substrates (Table 4). The half-life of boscalid in soil can be as high as 182 d (loamy sand soil) and as low as 9.7 d (grape field soil) [8,13]. The half-life of pyraclostrobin in soil can be 3.86–13.70 d [13,30]. In addition, the half-lives of boscalid were 1.90–2.01 d and 18.1–18.8 d, and the half-lives of pyraclostrobin were 1.62–1.73 d and 17.8–25.9 d, respectively, on greenhouse dill and grape [13,26]. Even the half-life of pyraclostrobin on cowpea reached 1.5–2.3 d [32]. The differences in dissipation behavior of these different substrates can be explained by climatic factors. Studies have shown that pesticides will show a faster dissipation rate in warm and humid environments, and high precipitation will undoubtedly increase the runoff risk of most compounds and promote the leaching potential of certain chemicals [33]. Long-term sunlight and high-temperature conditions will also promote the degradation and volatilization of pesticides [34]. In addition, higher temperatures can promote the growth of microorganisms and, to a certain extent, accelerate the degradation of pesticides by the environment [35]. In addition, acidification can be enhanced by high precipitation, and influence the degradation of pesticides [36]. Since Hunan province is in the subtropical monsoon climate zone, higher temperatures and abundant precipitation may cause the dissipation rate of fungicides to be different from that of other areas.

### 2.4. Residue Distributions of Boscalid and Pyraclostrobin in Watermelon

In six regions, after application at a rate of 270 g a.i./ha, the end residues of boscalid and pyraclostrobin in watermelon were detected. The quality control (QC) of real sample detection was performed in Table S3. The average recovery rate of boscalid and pyraclostrobin in watermelon was 86–105%, and the RSD was less than 9.1%. These data indicate that the detection method of boscalid and pyraclostrobin is stable and accurate, and the detection of field trial samples was reliable. The residual amount of boscalid and pyraclostrobin in the watermelon is lower than 0.05 mg/kg. Different berries, pome, and stone fruits exhibit a variety of shapes, sizes, and surfaces. Particularly, fruit size, volume–surface area relationship, and details of the skin structure likely influence the residue levels [37]. In previous studies, it was found that the residues of boscalid and pyraclostrobin in apples were at levels of 0.049 ± 0.007 mg/kg and 0.032 ± 0.003 mg/kg, respectively, which were similar to our results [38]. However, compared with grapes, watermelon has a smaller relative surface area and thicker skin and absorbs fewer pesticides. The residues in grapes were much higher, indicating that residues from pesticides are affected by the characteristics of the fruit itself, which requires a reasonable use plan to be designed based on the actual situation when using pesticides [13].

The MRLs of boscalid and pyraclostrobin of watermelons are 3 mg/kg and 0.5 mg/kg, respectively, according to the European Food Safety Authority (EFSA) [39,40]. Correspondingly, China proposes MRLs of 0.5 mg/kg for pyraclostrobin in watermelon to mitigate the health risks of human consumption [41]. However, no health guidance value for MRL is available for boscalid in watermelon in China. The terminal residues in watermelon in this present study were all far below the officially recommended values recorded above (Table S4). These results indicate that watermelon consumption is safe at 21 days after being sprayed with boscalid and pyraclostrobin at their recommended doses under open-field conditions. In addition, these results provide data for modifying the MRL values and the recommended dosage of these pesticides.

### 2.5. Dietary Exposure Risk Assessment

To evaluate consumer safety regarding pesticide residues, the exposure needs to be assessed and compared with health safety limits or toxicological endpoint values such as the acceptable daily intake and the acute reference dose [42]. The dietary risk probabilities of boscalid and pyraclostrobin in watermelon were assessed via RQ, which were calculated by comparing the value of the national estimated daily intake (NEDI) of topramezone with acceptable daily intake (ADI). Assessment of dietary risk to pesticide residues combines data on residues in foodstuff and data on food consumption. According to the above calculation method, the corresponding NEDI values calculated from the reference residue limit of the maximum dietary risk are 1.2187 mg and 1.1830 mg (Table 5), which is far less than the acceptable daily intake (ADI) established by the European Union. The supervised trials median residue (STMR) of boscalid and pyraclostrobin concluded from the field trials were both 0.05 mg/kg, the reference residue limit of evaluated watermelon. As shown in Table 5, the RQ of boscalid and pyraclostrobin were 48.4% and 62.6%, based on our residue data, ADI, and typical Chinese dietary intake patterns. High RQ values are indicative of higher levels of residual pesticide. In general, an RQ greater than 100% indicates an unacceptably high health risk to consumers [13]. Therefore, the above results demonstrate that applying boscalid and pyraclostrobin in watermelon fields with recommended dosage will not bring a significant potential dietary risk to Chinese consumers.

## 3. Materials and Methods

### 3.1. Reagents and Chemicals

The standards of boscalid (>98.6%) and pyraclostrobin (98.3%) were provided by the Shenyang Research Institute of Chemical Industry (Shengyang, China) and the Beijing Qinchengyixin Technology Co., Ltd. (Beijing, China), respectively. The 30% suspension concentrate (SC) commercial formulation of boscalid (20%) and pyraclostrobin (10%) applied on an open field was supplied by Jiangxi Xunzhong Agrochemical Co., Ltd. (Nanchang, China). The acetonitrile and formic acid of chromatographic grade were obtained from Mreda Technology Co., Ltd. (Beijing, China) and Beijing Dikma Co., Ltd. (Beijing, China), respectively. The cleanup sorbents, primary secondary amine (PSA, 40–60 μm), and graphitized carbon black (GCB, 120–400 mesh) were provided by Tianjin Bonna-Agela Technologies Venusil Technology Co., Ltd. (Tianjin, China). Anhydrous sodium chloride (NaCl) and magnesium sulfate (MgSO4) of analytical grade were obtained from Beijing Sinopharm Chemical Reagent Co., Ltd. (Beijing, China). Other analytical reagents were purchased from the Tianjin Jinke Research Institute of Meticulous Chemical Industry (Tianjin, China).

Standard stock solutions of individual boscalid and pyraclostrobin were prepared by weighing 25.3 mg and 12.7 mg, respectively, and by dissolving them in chromato-graphic grade acetonitrile. Mixed standard solutions were prepared by pipetting each standard solution into a 50 mL volumetric flask and then by filling it with chromatographic grade acetonitrile to a scaled value. The overall solutions were stored in the dark at 4 °C. 

### 3.2. Sample Pretreatment

The chopped watermelon samples were homogenized before pretreatment. Five grams of the homogenized samples were weighed into a 50 mL PTFE centrifuge tube. Three milliliters of water and ten milliliters of acetonitrile were added to the tube after standing for 30 min. The tube was vortexed for 1 min, and then, 1 g of sodium chloride and 4 g of anhydrous magnesium sulfate were added. After vortexing for 1 min again, the tube was centrifuged for 3 min at 2500 rpm. An amount of 1.5 mL of the supernatant acetonitrile phase was transferred into a new 5 mL centrifuge tube and cleaned up by a dispersive solid-phase extraction with 30 mg of PSA, 5 mg of GCB, and 150 mg of MgSO_4_. The extracts were vortexed for 30 s and then centrifuged for 3 min at 10,000 rpm. The supernatant was filtered through a 0.22 μm filter and transferred into an autosampler vial for HPLC-MS/MS analysis.

### 3.3. Instrumental Parameters

The Agilent HPLC–MS/MS system consisted of a 1200 Series liquid chromatography and a 6420 Triple Quad mass spectrometer equipped with an electrospray ionization source (ESI). An Agilent Zorbax SB-Aq C18 column (3.0 × 50 mm, 2.7 μm) was used to separate boscalid and pyraclostrobin and was maintained at 25 °C. The mobile phase was a mixture of 10% aqueous phase (0.2% formic acid in water) and 90% organic phase (pure acetonitrile) flowing at 400 μL/min. The column temperature was 25 °C. The injection volume was 5 μL.

The MS/MS acquisition parameters used for the quantification of the target compounds are provided in Table S1. The desolvation gas temperature was 350 °C, the desolvation gas flow was 10 L/min, and the nebulizer gas (N2) pressure was 45 psi. Analytes were determined in multiple reaction monitoring (MRM) mode.

### 3.4. Recovery Experiments

The standard solutions of boscalid and pyraclostrobin were added to control watermelon at the appropriate concentrations of 0.05, 3, and 10 mg/kg and of 0.05, 0.5, and 5 mg/kg, respectively. These samples were extracted and purified according to Section 3.2. Five parallel treatments for each sample were carried out. The accuracy and precision of the sample preparation method were evaluated by recoveries (80–120%) and relative standard deviation (RSD).

### 3.5. Field Trials

The supervised field experiments were designed according to NY/T 788–2018 issued by the Ministry of Agriculture, China. The open field trials were conducted in 2018 to observe the pesticide dissipation and residues in watermelon at six representative locations: Shandong province (temperate continental monsoon climate), Inner Mongolia (temperate continental monsoon climate), Ningxia (temperate continental monsoon climate), Guangxi (subtropical monsoon climate), Hunan (subtropical monsoon climate), and Shanxi (monsoon climate of medium latitudes). Each experiment plot area was 100 m^2^ with no application history of boscalid and pyraclostrobin. The use of other insecticides was forbidden on the plots during the trial period. The 30% boscalid and pyraclostrobin formulation was dissolved in water, and the aqueous solution was sprayed twice with internal pump backpack sprayers at an interval of 7 days. Each plot had three replications, and a buffer zone of 0.5 m was set to separate different plots. The untreated blocks of the same size were sprayed with water as the control. Samples were collected on the 7th, 10th, 14th and 21st day after spraying at least twelve fruits for each block. 

### 3.6. Statistical Analysis

The limits of quantifications (LOQs) were defined as the lowest spiked concentrations of target analytes in the matrix with a signal-to-noise ratio of 10 [23].

The dissipation rate and (t1/2) of boscalid or pyraclostrobin in watermelon were evaluated by subjecting the data to a first-order kinetics equation:

C_t_ = C_0_ e^−kt^ (1)

where C_t_ represents the concentration (mg/kg) of boscalid or pyraclostrobin residue at time (t), C_0_ represents the initial concentration (mg/kg) of boscalid or pyraclostrobin residue after application, and k is the dissipation coefficient on 1 day. The persistence of boscalid or pyraclostrobin is generally expressed in terms of t_1/2_ or DT50 ( i.e., the time of the disappearance of a pesticide to 50% of its initial concentration) and was calculated from the k value as follows:

t_1/2_ = ln2/k (2)

### 3.7. Dietary Risk Assessment

The national estimated daily intake (NEDI) of boscalid or pyraclostrobin and the risk quotient (RQ) were calculated using the following formulas:

(3) $$\mathrm{NEDI}=\sum^{}\frac{STMR_{i}}{STMR-P_{i}}\times F_{i}$$

RQ = NEDI/ADI × bw(4)

where *STMR_i_* (mg/kg) was the supervised trials’ median residue of boscalid or pyraclostrobin in certain kinds of food registered by China; *F_i_* (kg) was the dietary reference intake for certain kinds of food used to plan and assess nutrient intakes of healthy Chinese people; ADI (mg/kg bw) was the acceptable daily intake of boscalid or pyraclostrobin, and bw was the average body weight of a Chinese adult (63 kg). If there was no suitable *STMR_i_*, the corresponding MRLs would be used for the NEDI calculation.

## 4. Conclusions

A simple, sensitive, and efficient method incorporating a modified QuEChERS technique and HPLC-MS/MS detection was developed and evaluated for simultaneous quantitation of boscalid and pyraclostrobin levels in watermelon. This method exhibited satisfactory performance regarding linearity, accuracy, and reproducibility. The method provides a guarantee for the monitoring of fungicides in watermelon in large quantities with lower costs and a faster detection time. Both boscalid and pyraclostrobin in watermelon degraded rapidly under field-incurred conditions. The results of the dietary risk assessments in watermelon yielded RQs below 100%. These data show that the potential health risks imposed by ingesting these fungicides in watermelon were not significant. This study provides guidance on the reasonable use of boscalid and pyraclostrobin in a watermelon ecosystem, as well as data for the modification of the MRL values and the recommended dosage of these pesticides.

## Figures and Tables

**Table 1:** Recoveries (*n* = 5) of boscalid and pyraclostrobin in different matrices.

Compounds	Spiked Level (mg/kg)	Watermelon	
		Average Recoveries (%) *n* = 5	RSD (%)
Boscalid	0.05	108	5.4
	3	101	7.7
	10	97	7.8
Pyraclostrobin	0.05	103	2.5
	0.5	97	7.3
	5	93	9.1

“*n* = 5” represents five repetitions for each level.

**Table 2:** Comparison of detection methods of boscalid and pyraclostrobin in different matrices.

Compounds	Matrix	Method	LOQ (mg/kg)	Retention Times (mins)	Reference
Boscalid and Pyraclostrobin	Watermelon	LC–MS/MS	0.01,0.005	0.8–0.9	This study
	Grape	RRLC–MS/MS	0.001	0.80, 0.97	[13]
	Greenhouse dill	LC–MS/MS	0.001	13.19, 14.60	[26]
Boscalid	Honeybees	UHPLC–QqQ	0.0001	6.50	[27]
	Grape pulp	ACSV *	2.73	No data	[28]
	Persimmon	LC–MS/MS	0.001	4.59	[29]
Pyraclostrobin	Rosa roxburghii	LC–MS/MS	0.00024	5.00	[30]
	Fritillaria	LC–MS/MS	0.01	1.98	[31]
	Cowpea	LC–MS/MS	0.01	No data	[32]

* ACSV is an acronym for Adsorptive Cathodic Stripping Voltammetry.

**Table 3:** Dissipation data of boscalid and pyraclostrobin in watermelon in Hunan province.

Location	Dose (g a.i./ha)	Spray Times	Intervals (d)	Average Residue (mg/kg) *n* = 3 *	
				Boscalid	Pyraclostrobin
Hunan	270	2	7	0.5323	0.2938
			10	0.1455	0.0505
			14	0.0704	0.0225
			21	0.0118	0.0044
			28	0.0083	0.0027

* “*n* = 3” represents three repetitions for each detection.

**Table 4:** Comparison of half-lives of boscalid and pyraclostrobin in different matrices.

Compounds	Matrix	Half-Lives (DT50, Day)	Reference
Boscalid	Loamy sand soil	104–182	[8]
	Greenhouse dill	1.90–2.01	[26]
	Topsoil from dill cultivation	2.64–4.85	[26]
	Grape	18.1–18.8	[13]
	Grape field soil	9.7–17.6	[13]
	Watermelon	3.52	This study
Pyraclostrobin	Greenhouse dill	1.62–1.73	[26]
	Topsoil from dill cultivation	2.08–2.11	[26]
	Grape	17.8–25.9	[13]
	Grape field soil	8.9–13.7	[13]
	Rosa roxburghii	6.20–7.79	[30]
	Rosa roxburghii soil	3.86–5.95	[30]
	Fritillaria	6.3	[31]
	Cowpea	1.5–2.3	[32]
	Watermelon	3.27	This study

**Table 5:** The chronic dietary intake risk assessment of boscalid and pyraclostrobin in accordance with Chinese dietary patterns.

Food Classification	Fi (kg)	Boscalid		Pyraclostrobin	
		Reference Residue Limits or STMR (mg/kg)	NEDI (mg)	Reference Residue Limits or STMR (mg/kg)	NEDI (mg)
Rice and its products	0.2399			0.02 (EU)	0.004798
Flour and its products	0.1385			0.2 (CN)	0.0277
Other grains	0.0233			0.02 (CAC)	0.000466
Tubers	0.0495	1 (CN)	0.0495	0.2 (CN)	0.0099
Dried beans and their products	0.016				
Dark vegetables	0.0915	2 (CN)	0.183	1 (CN)	0.0915
Light vegetable	0.1837	5 (CN)	0.9185	5 (CN)	0.9185
Pickles	0.0103				
Fruits	0.0457	0.05 (STMR)	0.002285	0.05 (STMR)	0.002285
Nuts	0.0039				
Livestock and poultry	0.0795				
Milk and its products	0.0263				
Egg and its products	0.0236				
Fish and shrimp	0.0301				
Vegetable oil	0.0327	2 (CN)	0.0654	0.2 (CN)	0.00654
Animal oil	0.0087				
Sugar, starch	0.0044				
Salt	0.012			10 (CN)	0.12
Soy sauce	0.009			0.15 (CAC)	0.00135
Total	1.0286		1.2187		1.1830
ADI × 63 (mg)			2.52		1.89
Risk quotient (%)			48.4		62.6

STMR_i_ (mg/kg) represented supervised trials median residue of tembotrione in maize in China, Fi referred to the daily intake of a certain agricultural product or food in China (kg), and bw was the mean of average body weight of Chinese adult (63 kg). CN: China; EU: European Union; CAC: Codex Alimentarius Commission.

## Data Availability

The data are contained within the article.

## Supplementary Materials

The following supporting information can be downloaded at https://www.mdpi.com/article/10.3390/molecules27144410/s1. Table S1: The MRM acquisition parameters; Table S2: Calibration curves and matrix effects of pesticides; Table S3: Quality control (QC) of real sample detection; Table S4: Terminal residues of boscalid and pyraclostrobin in watermelon samples; Figure S1: LOQ determination of boscalid and pyraclostrobin; Figure S2: Mass spectra of boscalid and pyraclostrobin in MRM mode.

## Sample Availability

Samples of the compounds boscalid and pyraclostrobin are available from the authors.
