# Supplementary material for: Dissipation Residue Behaviors and Dietary Risk Assessment of Boscalid and Pyraclostrobin in Watermelon by HPLC-MS/MS
Source: Molecules. 2022 Jul 9;27(14):4410. doi: 10.3390/molecules27144410 (PMC9318032; doi:10.3390/molecules27144410)
Supplement: Supplementary file 1 [file molecules-27-04410-s001.zip › molecules-1782607-supplementary.pdf]

Supplementary

Dissipation Residue Behaviors and Dietary Risk Assessment of Boscalid and Pyraclostrobin in Watermelon by HPLC-MS/MS

Le Lv †, Yue Su †, Bizhang Dong, Wang Lu, Jiye Hu and Xiaolu Liu *

School of Chemistry and Biological Engineering, University of Science and Technology Beijing, Beijing 100083, China; lvle@ustb.edu.cn (L.L.); suyue_2022@163.com (Y.S.); dongbizhang@ustb.edu.cn (B.D.); 18810861030@163.com (W.L.); jyhu@ustb.edu.cn (J.H.)

* Correspondence: xiaoluliu@ustb.edu.cn

† These authors contributed equally to this work.

Table S1. The MRM acquisition parameters

Compounds	Retention time (min)	Precursor ion (m/z)	Quantitative ion (m/z)	Qualitative ion (m/z)	Fragmentor (V)	Collision energy (eV)
boscalid	0.8	342.9	306.8	139.9	135	306.8/20<br>139.9/20

pyraclostrobin	0.9	387.9	194	163	88	194/8
						163/24

Table S2. Calibration curvesof pesticides in watermelon matrices.

Matrix	Compounds	Linear ranges (mg/kg)	Calibration curves	Correlation coefficient (r)
Watermelon	Boscalid	0.01-10	y = 8872.6x + 384.09	0.9993
	Pyraclostrobin	0.005-5	y = 122042x + 16272	0.9952

x and y were defined as the absolute injection volume and average peak area of quantitative ion, respectively.

Table S3. Quality control (QC) of real sample detection

Matrix	Date of detected	Spiked level (mg/kg)	Compounds	Average recovery (%)	RSD (%)
Watermelon	2019.4.27	0.05	Boscalid	105	6.2
			Pyraclostrobin	86	9.1

Table S4. Terminal residues of boscalid and pyraclostrobin in watermelon samples

Location	Dose (g a.i. /ha)	Spray times	Intervals (d)	Terminal residue (mg/kg)	
				Boscalid	Pyraclostrobin
Laiyang city of Shandong province	270	2	14	<0.05	<0.05
			21	<0.05	<0.05
Wulanchabu city of Inner Mongolia	270	2	14	<0.05	<0.05
			21	<0.05	<0.05
Yinchuan city of Ningxia province	270	2	14	<0.05	<0.05
			21	<0.05	<0.05
Yulin city of Guangxi province	270	2	14	<0.05	<0.05
			21	<0.05	<0.05
Yueyang city of Hunan province	270	2	14	0.062	<0.05
			21	<0.05	<0.05

Xianyang city of Shanxi	270	2	14	<0.05	<0.05
province			21	<0.05	<0.05

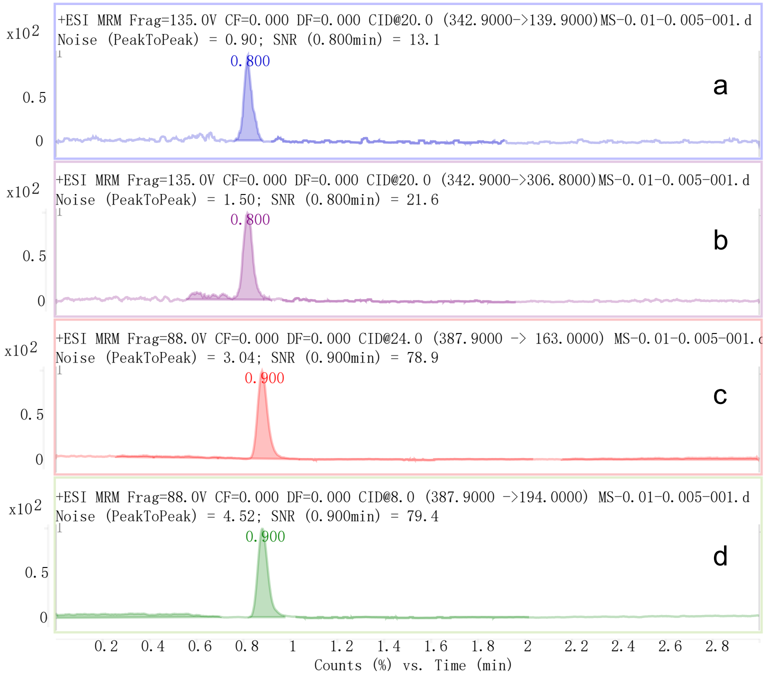

Figure S1. LOQ determination of boscalid and pyraclostrobin. The limits of quantifications (LOQs) were defined as the lowest spiked concentrations of target analytes in the matrix with a signal-to-noise ratio of 10. a) and b) represent the signal-to-noise ratio of boscalid qualitative and quantitative ions in MRM mode, respectively; c) and d) represent the signal-to-noise ratio of pyraclostrobin qualitatively and quantitatively in MRM mode, respectively.

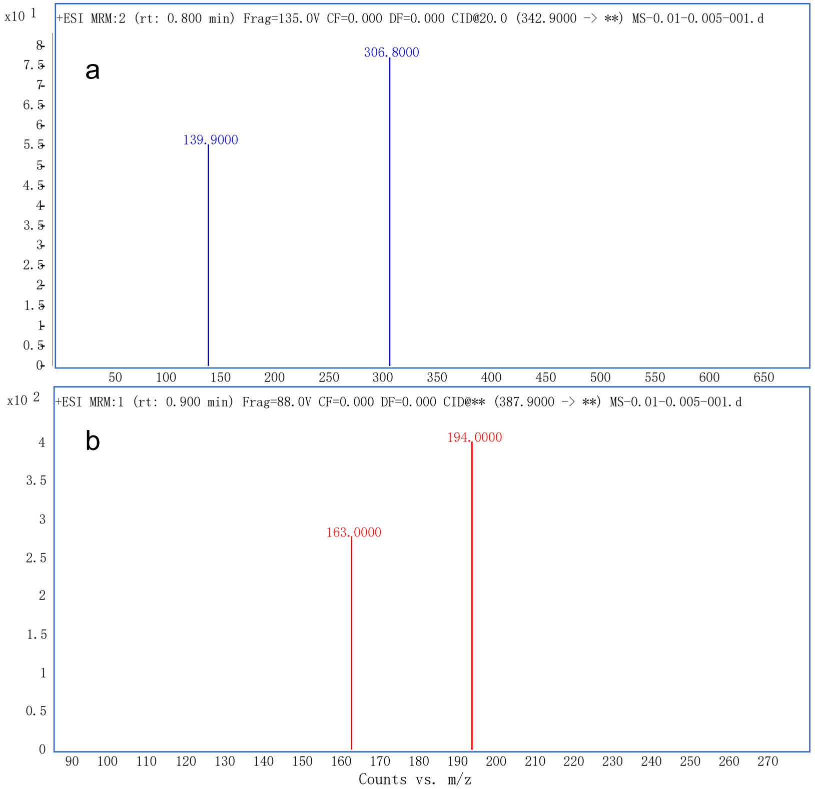

Figure S2. Mass spectra of boscalid and pyraclostrobin in MRM mode. a) Represents the qualitative (m/z = 139.9) and quantitative (m/z = 306.8) ions of boscalid); b) represents the qualitative ion (m/z=163) and the quantitative ion (m/z=194) of pyraclostrobin.
